# Physical activity and cardiovascular disease risk factors among young and middle-aged men in urban Mwanza, Tanzania

**Published:** 2012-01-20

**Authors:** Alfa Muhihi, Marina Njelekela, Rose Mpembeni, Zablon Masesa, Kazuya Kitamori, Mari Mori, Norihiro Kato, Jacob Mtabaji, Yukio Yamori

**Affiliations:** 1 Harvard School of Public Health, Boston, Massachusetts 02115, United States; 2 Department of Physiology, Muhimbili University of Health and Allied Sciences, Dar es Salaam, Tanzania; 3 Department of Physiology, Catholic University of Health and Allied Sciences - Bugando, Mwanza, Tanzania; 4 Department of Food and Nutritional Environment, Human Life and Environment, Kinjo Gakuin University, Nagoya, Japan; 5 Institute for World Health Development, Mukogawa Women's University, Nishinomiya, Japan; 6 Department of Gene Diagnostics and Therapeutic Research Institute, International Medical Center of Japan, Tokyo, Japan

**Keywords:** Urbanization, Nutrition transition, Physical activity energy expenditure, Occupation intensity, Tanzania

## Abstract

**Background:**

Cardiovascular diseases (CVD) risk factors are increasing at an unprecedented rate in developing countries. However, fewer studies have evaluated the role of physical activity in preventing CVD in these countries. We assessed level physical activity and its relationship with CVD risk factors among young and middle aged men in a fast growing city of Mwanza in Tanzania.

**Methods:**

Physical activity was assessed among 97 healthy men aged 20–50years using Sub-Saharan Africa Activity Questionnaire. An updated compendium of physical activity was used to code the metabolic equivalent. Energy expenditure was calculated using Harris Benedict equation. Anthropometric measurements, blood pressure, fasting blood glucose and serum lipids were also measured.

**Results:**

The mean energy expenditure in this population was 6,466±252 kcal/week. More than half (53.6%) of the participants had energy expenditure of ≥4,000 kcal/week. Only three (3.1%) had energy expenditure below the recommended 1,000 kcal/week. Except for hypertension, prevalence of CVD risk factors was low in this population; hypertension 23.7%, low HDL-cholesterol 10.3%, high LDL-cholesterol 9.3% and obesity 4.1%. Physical activity energy expenditure had an inversely relationship with waist to hip ratio, systolic blood pressure, heart rate, total cholesterol, HDL-cholesterol, LDL-cholesterol, triglycerides and fasting blood glucose.

**Conclusion:**

Physical activity energy expenditure was high in this population and was inversely correlated with CVD risk factors. Physical activity may play an important role in the prevention of CVD in this urban population of young and middle aged men.

## Background

Physical activity is any bodily movement produced by skeletal muscles and results in energy expenditure beyond the basal energy expenditure [1]. Levels of physical activity vary considerably from person to person and over time, but all members of the community exhibit some physical activity.

Physical activity provides many health benefits including prevention of many chronic diseases [2]. People who are physically active have reduced risk of developing or dying from heart disease, diabetes and hypertension and outlive those who are inactive [3]. Physical inactivity has been associated with increasing prevalence of cardiovascular diseases, with most recent estimates suggesting that almost 2 million deaths per year worldwide are attributable to physical inactivity [4].

Developed countries like United States, have guidelines for physical activity recommending all young people to participate in physical activity of at least moderate intensity for one hour per day [5]. World Health Organization has also set recommendations for physical activity for both developed and developing countries [6]. The International prevalence study on physical activity in twenty countries [7] found the prevalence of low physical activity ranging from 7% to 43% and from 6% to 49% among men and women respectively. It also showed significant variation in physical activity across countries.

Energy expenditure related to physical activity has been reported to be higher among African than Western populations [6]. Several factors like infrastructure and culture that supports walking are known to influence the level of physical exercise [7]. Other factors like age, body mass index, personal attitude towards body shape, perceived peer attitude about body shape/fitness as well as parental attitude about physical activity are associated with physical activity among adolescents [8].

Urban-rural difference in physical activity exists among Africans [6]. The rural children in the Nandi region of Kenya were found to spend significantly more time doing moderate to vigorous physical activities compared to urban children [9]. The prevalence and incidence of CVD risk factors and various cardiovascular disorders in African populations also show an urban-rural difference [10]. Whether such urban-rural gradient is attributable to physical activity has not yet been defined clearly. In Tanzania, the Masai have been shown to have highest level of physical activity than rural Bantu and urban dwellers [11]. However, the level of physical activity and its relationship with CVD risk factors has not been fully explored. This cross sectional study assessed the level of physical activity and its relationship with CVD risk factors among young and middle aged men in urban Mwanza, Tanzania.

## Methods

This survey was conducted in four administrative wards of Kirumba, Nyakato, Igoma and Mkolani in urban Mwanza, the second largest city in Tanzania, located on the shore of Lake Victoria. Mwanza urban has two districts namely Nyamagana and Ilemela. It also has two divisions which are administratively further divided into wards and streets. In total, Mwanza urban has 21 wards. Of the four wards in which this survey was conducted, Kirumba, Nyakato and Igoma are purely urban wards while Mkolani is a semi-urban ward. Kirumba, Nyakato and Igoma wards are with 5 kilometer radius from the city center while Mkolani is more than 10 kilometers from the city center. Two streets were randomly selected for each ward, and a list of men aged 20-50years was obtained from heads of the selected streets. All the obtained data were computerized, and a random list of 35 participants for each ward was generated. A total of 140 men were invited to participate into the study. The refusal rate was 31% mainly due to fear of HIV testing from the blood samples which were collected for analysis of serum lipids (verbal communication). The study was approved by the ethical committees of Weill Bugando University College of Health and Allies Sciences and Mukogawa Women's University, and a written informed consent was obtained from all participants before being enrolled into the study.

Data collection procedures for this study have already been described elsewhere [12]. Data was collected in accordance with the basic protocol of the WHO-CARDIAC (Cardiovascular Disease and Alimentary Comparison) Study. This was a WHO coordinated multi-center epidemiological survey on dietary habits and CVD risk factors and mortalities in 61 populations from various countries. Briefly, all measurements were conducted by an experienced physician and one nurse at a local health care facility. Body weight and height were measured with subjects standing and wearing light clothing and without shoes. Weight was recorded to the nearest 0.1kg, while height was measured to the nearest 0.5cm using a portable stadiometer. Body Mass Index (BMI) was then calculated as weight in kilograms divided by height in meters square (kg/m^2^). Overall obesity was defined as BMI of >30kg/m^2^ [13]. Waist and hip circumferences (WC and HC) were measured to the nearest 0.5cm using a flexible tape measure. Waist circumference of >102 cm was used to define central obesity in this population. Waist to hip ratio (WHR) was calculated as WC divided by HC and a high WHR was defined as ≥0.9 [13].

Blood pressure and pulse rate were measured after 5 to 10 minutes of rest using an automatic digital blood pressure measurement system (Omron Digital HEM-907, Tokyo, Japan). Three readings were taken for each participant, and an average of the three readings was used in the final analysis. Hypertension was defined using the World Health Organization and International Society of Hypertension (WHO/ISH) guidelines on hypertension [14].

Venous blood samples were collected in the morning upon participant's arrival at the study site following at least 10 hours of fasting, and were separated within 6 to 8 hours of collection before being frozen at minus 40 degrees centigrade. All samples were transported under standard conditions using liquid nitrogen to a central laboratory at the Institute of World Health Development, Mukogawa Women's University, Nishinomiya, Japan for analysis. Total cholesterol (TC), triglycerides (TG), high density lipoprotein cholesterol (HDL-C) and fasting blood glucose (FBG) were analyzed at SRL Inc. (Tokyo, Japan). Enzymatic methods for TC, HDL-C, TG and FBG were performed [12]. LDL-C was estimated using the Fried-Wald formula (i.e., LDL=Total cholesterol-HDL-(TG/5)) [15]. High TC was defined as ≥6.2 mmol/L, high LDL-C as ≥3.8 mmol/L, low HDL-C as<1.0 mmol/L and high TG as ≥1.7mmo/L [16]. Diabetes was defined as fasting blood glucose level of ≥7.0mmol/L [17].

Assessment of physical activity was done using a validated Sub-Saharan Africa Activity Questionnaire (SSAAQ) for assessment of physical activity in epidemiological studies [18]. Frequency, duration, and intensity were measured for each reported physical activity and was coded into Metabolic Equivalent (METs) using updated compendium of physical activity [19]. Based on METs, participant's main occupations were grouped as light (6.0METs)[20]. Physical activity energy expenditure was calculated as Kcal/week using the Harris Benedict equation. We used the 75^th^ percentile of the distribution of physical activity energy expenditure per week as a cut-off for the comparison of significant differences in the prevalence of physical activity. Participants who had physical activity energy expenditure of ≥4000kcal/week were considered as having higher physical activity energy expenditure.

Socioeconomic status was assessed using a pre-tested structured questionnaire. Level of education of the participants was assessed and was classified into two for analysis; Up to 7 years of schooling (Primary education or less) and more than 7 years of schooling (Secondary education and above). Monthly income in Tanzanian shillings (Tshs) was categorized into two levels; ≤50,000 Tanzanian shillings (≤35 US$) and>50,000 Tanzanian shillings (>35 US$) based on the overall income of young adults in Mwanza. Cigarette smoking was defined as smoking at least one cigarette per day for a year [21]. Smoking was categorized as never smoker, current smoker and former smoker. Alcohol consumption (frequency and amount) were also evaluated.

All the data were entered in an excel spread sheet and were then imported into a Statistical Analysis Software version 9.2 (SAS Institute Inc., North Carolina, USA) for analysis. Prevalence of CVD risk factors by energy expenditures as well as physical activity and socioeconomic variables were compared using the chi-square test. Pearson's correlation coefficient was used to assess the relationships among cardiovascular disease risk factors and energy expenditure. Results are presented as means±standard deviations. In all the analyses, P<0.05 was considered statistically significant.

## Results

The mean age for the participants was 31.6±6.4 years. Socio-demographic characteristics of the study population are demonstrated in Table 1. More than half (51.6%) were single and 35% were married. More participants (68%) had primary level of education or less, were involved in manual works (45.4%) and had low monthly income (72.2%). Fifty two participants (53.6%) reported drinking alcohol whereas as 15 (15.5%) were smoking.

**Table 1:** Sociodemographic characteristics of the study population

Characteristic	Number (N)	Percentage (%)
Age (Yrs) (Mean ±Sd)	31.6±6.4	**-**
**Marital status (N=97)**		
Single	50	51.6
Married	34	35.0
Other*	13	13.4
**Years of education (N=97)**		
≤7 years	66	68.0
> 7 years	31	32.0
**Ward of residence (N=97)**		
Kirumba	24	24.8
Nyakato	7	7.2
Igoma	29	29.9
Mkolani	37	38.1
**Occupation (N=97)**		
Not working	7	7.2
Non-manual work	17	17.5
Manual work	44	45.4
Subsistence farmer (Owner)	11	11.3
Subsistence farmer (Employee)	18	18.6
Income/Month^**^(N=97)		
Tshs ≤50,000	70	72.2
Tshs>50,000	27	27.8
**Drink alcohol (N=97)**		
Yes	52	53.6
No	45	46.4
**Smoking (N=97)**		
Yes	15	15.5
No	82	84.5

* Other marital statuses were; Divorced (7), Separated (2), Widowed (2) and Cohabiting (2)

Majority (78.9%) of the participants were involved in moderate intensity occupations. None of the participants reported to be involved in intense occupation. The median physical activity energy expenditure was 4,347kcal/week (IQR 1918-9422 kcal/week). More than half (53.6%) of the participants had energy expenditure of 4,000 Kcal/week or more. Walking was the commonest means of getting to work for majority (83.3%) of the participants (Table 2).

**Table 2:** Level of physical activity among young and middle-aged men in urban Mwanza, Tanzania

Characteristic	Number (N)	Percentage (%)
**Occupation Intensity (METs) (N= 90)**		
Light (METs<3)	19	21.1
Moderate (METs=3-6)	71	78.9
**Occupation (#MET-hour/day) (N=90)**		
Less than 26	28	31.1
26 to 37	34	37.8
More than 37	28	31.1
**Energy expenditure (kcal/week) (N=97)**		
≤4,000	45	46.4
>4,000	52	53.6
**Mode of getting to work**†**(N=90)**		
Walking	75	83.3
Bicycle	5	5.6
Car	10	11.1
**Self-Work Evaluation (N=97)**		
Light	4	4.2
Moderate	40	41.2
Heavy	53	54.6

^†^ Seven (7) participants who reported to have no occupation had missing data on mode of getting to work

Participants who were more than 30 years old were involved in moderate intensity occupations than the young ones (P=0.006). There was no difference in occupation intensity with regard to level of education and income. Overall physical activity energy expenditure did not show any significantly association with age, level of education or income (Table 3). The prevalence of CVD risk factors by energy expenditure are shown in Table 4. No significant difference was observed in the prevalence of CVD risk factors between those with energy expenditure of <4,000 kcal/week and those with energy expenditure of ≥4,000 kcal/week. Except for hypertension, overall prevalence of CVD risk factors was found to be low in this population; hypertension was found to be 23.7%, low HDL-cholesterol 10.3%, high LDL-cholesterol 9.3%, obesity 4.1%, hypercholesterolemia 4.1% and hypertriglyceridemia 1.0%. Only three participants (3.1%) had metabolic syndrome. None of the participants was found to have diabetes (results not shown).

**Table 3:** Relationship between socioeconomic and demographic factors with occupation intensity and physical activity energy expenditure

	Occupation intensity		
		
Characteristic	Light (< 3 METs)	Moderate (3-6 METs)	P-value
**Age (N=97)**			
≤30 years	16 (76.2%)	28 (36.8%)	0.006
>30 years	5 (23.8%)	48 (63.2%)
**Years of education (N=97)**			
≤7 years	14 (66.7%)	52 (68.4%)	0.879
> 7 years	7 (33.3%)	24 (31.6%)
**Monthly Income**^a^**(N=97)**			
≤ Tshs 50,000/=	17 (80.9%)	52 (68.4%)	0.262
> Tshs 50,000/=	4 (19.1%)	24 (31.6%)
**Physical activity energy expenditure**			
	**< 4,000Kcal/week**	**≥4,000Kcal/week**	
**Age (N=97)**			
≤30 years	22 (48.9%)	26 (50.0%)	0.913
>30 years	23 (51.1%)	26 (50.0%)
**Years of education (N=97)**			
≤7 years	32 (71.1%)	34 (65.4%)	0.546
> 7 years	13 (28.9%)	18 (34.6%)
**Monthly Income^a^ (N=97)**			
≤Tshs 50,000/=	33 (73.3%)	36 (69.2%)	0.657
>Tshs 50,000/=	12 (26.7%)	16 (30.8%)

^a^1 US Dollar=1460 Tanzanian Shillings (Tshs)

**Table 4:** Prevalence of CVD risk factors by physical activity energy expenditure among young and middle-aged men in urban Mwanza, Tanzania

	Physical activity energy expenditure		
		
CVD risk factors	<4,000Kcal/week	≥4,000Kcal/week	P-value
**Hypertension (BP≥140/90mmHg)**			
High	14 (31.1%)	9 (17.3%)	0.09
Normal	31 (68.9%)	43 (82.7%)
**Overall Obesity (BMI≥30kg/m**^**2**^**)**			
Obese	1 (2.2%)	3 (5.8%)	0.36
Non-obese	44 (97.8%)	49 (94.2%)
**Central Obesity (WC>102cm)**			
Obese	2 (4.4%)	2 (3.8%)	0.64
No-obese	43 (95.6%)	50 (96.2%)
**Hypercholesterolemia (≥6.2mmol/L)**			
High	2 (4.5%)	2 (3.8%)	0.63
Normal	42 (95.5%)	50 (96.2%)
**Elevated LDL-C (≥ 3.8mmol/L)**			
High	5 (11.4%)	4 (7.7%)	0.39
Normal	39 (88.6%)	48 (92.3%)
**Low HDL-C (<1.0mmol/L)**			
Low	3 (6.8%)	7 (13.5%)	0.24
Normal	41 (93.2%)	45(86.5%)
**Elevated TG (≥1.7mmol/L)**			
High	1 (2.3%)	0 (0.0%)	0.46
Normal	42 (97.7%)	51 (100%)

BP=Blood pressure; BMI=Body mass index; WC=Waist circumference; LDL-C=Low density lipoprotein cholesterol; HDL-C=High density lipoprotein cholesterol; TG=Triglycerides

The correlations between physical activity energy expenditure are presented in Table 5. Physical activity energy expenditure had a significant inverse association with systolic blood pressure (r=0.20, p=0.04). Although it was not significant, physical activity energy expenditure also had an inverse correlation with waist to hip ratio, diastolic blood pressure, heart rate, total cholesterol, HDL-cholesterol, LDL-cholesterol, triglycerides and fasting blood glucose. In short, higher physical activity energy expenditure was associated with lower CVD risk factor profile. Contrary to what was expected (positive association); HDL-cholesterol was also inversely correlated with physical activity energy expenditure.

**Table 5:** Pearson's correlation coefficients *(r)* between CVD risk factors and physical activity energy expenditure<br><br>

CVD Risk Factors	Energy Expenditure (Kcal/Week)	P-value
BMI (kg/m^2^)	0.08	0.42
WC (cm)	0.05	0.64
Waist to Hip Ratio	−0.12	0.25
Systolic BP (mmHg)	−0.20	0.05
Diastolic BP (mmHg)	−0.13	0.21
Heart Rate (beats/min)	−0.18	0.07
TC (mmol/L)	−0.07	0.52
LDL-C (mmol/L)	−0.02	0.81
HDL-C (mmol/L)	−0.09	0.38
TG (mmol/L)	−0.05	0.62
FBG (mmol/L)	−0.11	0.31

Values are presented as Pearson's correlation coefficients (*r*); BMI=Body mass index; WC=Waist circumference; TC=Total cholesterol; LDL-C=Low density lipoprotein cholesterol; HDL-C=High density lipoprotein cholesterol; TG=Triglycerides; FBG=Fasting blood glucose

## Discussion

This population of young and middle aged men in an urban setting of Mwanza has demonstrated an overall high level of physical activity energy expenditure. Physical activity energy expenditure was inversely associated with CVD risk factors. Urban Tanzanians have been reported to have considerably lower physical activity level and a more unfavorable lipid pattern than rural Tanzanians [11]. A high level of physical activity has previously been reported among the rural Masai (Mean=2,565 kcal/day) while the urban Bantu had an energy expenditure of 891 kcal/day [11]. Several other studies have reported urban residents to be less physically active compared to their counter-parts rural dwellers [22].

The influence of socioeconomic status on physical activity is well known. Physical activity tends to decrease with increasing age [7]. In our study, we did not find any significant association between energy expenditure with the socioeconomic status of the participants. Middle aged men were involved more in moderate intense occupations compared to the young ones. In developing countries like Tanzania, people with low socioeconomic status are the ones who are more physically active as they have to do more manual work for their daily living compared to those with high socioeconomic status. The lack of association between socioeconomic status and physical activity energy expenditure in our study could be explained by the fact that majority of the participants were young with low socioeconomic status and were mainly involved in manual works. Similar findings were reported in Scotland where they failed to demonstrate significant difference in physical activity between affluent and the deprived participants [23].

In our study, we did not find a strong correlation between energy expenditure with CVD risk factors. The correlation was however in the expected direction (negative association), but again contrary to what was expected, the HDL-cholesterol was also in the negative direction. Hayes et al also reported non-significant negative association between physical activity index and cardiovascular disease and diabetes risk markers among men but was significant among women [24]. Other studies have also documented the difficulties in demonstrating the relationship between physical activity and biological measurements like serum lipids [25]. The main reason suggested is the size of the error that is generated in the measurement of complex behaviors like physical activity using a questionnaire [24]. The physical activity levels obtained from questionnaires only gives the estimate and cannot be relied upon.

Being fit or active has been shown to be associated with a greater than 50% reduction in the risk of CVD [26]. All men in this study except for three, had energy expenditure of more than 1,000 Kcal/week. It has been shown that an energy expenditure of 1,000Kcal/week decreases the risk of non-communicable disease, decrease mortality and increase life expectancy [20].

Several biological mechanisms through which physical activity decreases the risk for cardiovascular diseases have been postulated. Routine physical activity has been shown to improve abdominal obesity and weight control [27], glucose homeostasis and insulin sensitivity [6], coronary blood flow and reduces blood pressure [20]. It also improves lipid lipoprotein profile (reduced triglyceride levels, increased high density lipoprotein cholesterol and reduced low density lipoprotein cholesterol to high density lipoprotein cholesterol ratios) [28]. The present study has several limitations. Firstly, it was a cross-sectional study, which does not allow any inference to be drawn with regard to the causal relationship. Secondly, the study comprised of a relatively small representative sample of men, residents of Mwanza city and may therefore suffer statistical power to detect true differences between groups. Nevertheless, this study provides useful information on the prevalence of CVD risk factors and their socioeconomic and demographic correlates among young and middle aged men in urban Mwanza.

## Conclusion

This population has demonstrated high level of physical activity and low profile of CVD risk factors. Physical activity energy expenditure was inversely correlated with CVD risk factors. Physical activity is an appropriate and cost effective intervention for primary prevention of CVD. In order to maintain such a low level of CVD risk factors, this population should maintain such a high level of physical activity as urbanization and nutrition transition take their course. A larger study comprising of both men and women is recommended to better characterize physical activity and its relationship with CVD risk factors.
